# Radiation intensity (${\text{CTDI}}_{\text{vol}}$) and visibility of anatomical structures in head CT examinations

**DOI:** 10.1120/jacmp.v17i1.5701

**Published:** 2016-01-08

**Authors:** Sameer Tipnis, Rajesh Thampy, Zoran Rumboldt, Maria Spampinato, Gisele Matheus, Walter Huda

**Affiliations:** ^1^ Department of Radiology and Radiological Sciences Medical University of South Carolina Charleston SC USA

**Keywords:** head CT, CTDI_vol_, basal ganglia, fourth ventricle, observer study, optimization

## Abstract

The purpose of this study was to quantify how changing the amount of radiation used to perform routine head CT examinations (${\text{CTDI}}_{\text{vol}}$) affects visibility of key anatomical structures. Eight routine noncontrast head CT exams were selected from six CT scanners, each of which had a different ${\text{CTDI}}_{\text{vol}}$ setting (60 to 75 mGy). All exams were normal and two slices were selected for evaluation, one at the level of basal ganglia and the other at the fourth ventricle. Three experienced neuroradiologists evaluated the visibility of selected structures, including the putamen, caudate nucleus, thalamus, internal capsule, grey/white differentiation, and brainstem. Images were scored on a five‐point scoring scheme (1, unacceptable, 3, satisfactory, and 5, excellent). Reader scores, averaged over the cases obtained from each scanner, were plotted as a function of the corresponding ${\text{CTDI}}_{\text{vol}}$. Average scores for the fourth ventricle were 3.06±0.83 and for the basal ganglia were 3.20±0.86. No image received a score of 1. Two readers showed no clear trend of an increasing score with increasing ${\text{CTDI}}_{\text{vol}}$. One reader showed a slight trend of increasing score with increasing ${\text{CTDI}}_{\text{vol}}$, but the increase in score from a 25% increase in ${\text{CTDI}}_{\text{vol}}$ was a fraction of the standard deviation associated average scores. Collectively, results indicated that there were no clear improvements in visualizing neuroanatomy when ${\text{CTDI}}_{\text{vol}}$ increased from 60 to 75 mGy in routine head CT examinations. Our study showed no apparent benefit of using more than 60 mGy when performing routine noncontrast head CT examinations.

PACS number(s): 87.57.C‐

## I. INTRODUCTION

For any CT examination, the operator has to choose the quality of the X‐ray beam as well as the corresponding quantity. X‐ray beam quality is determined by the X‐ray tube voltage and beam filtration, and is normally expressed as a half value layer (HVL) of aluminum (Al). In CT, with typical HVL values of about 8 mm Al, changing the beam quality influences image contrast, particularly for high atomic number structures such as bone and iodine. X‐ray beam intensity (quantity) is normally specified in terms of the volume CTDI (${\text{CTDI}}_{\text{vol}}$), and is controlled by the choice of the X‐ray tube current (mA), the X‐ray tube rotation time(s), as well as the pitch when scans are performed in helical mode.^(1)^ For a given patient, choice of ${\text{CTDI}}_{\text{vol}}$ will primarily influence the amount of mottle (noise) in the resultant CT image.^2^, ^3^ Ideally, the choice of X‐ray beam quality (kV) and quantity (${\text{CTDI}}_{\text{vol}}$) in any CT examination should be (just) sufficient to ensure that diagnostic performance is satisfactory. It is therefore incumbent on the operator to decide on the acceptable level of mottle, and thereby the ${\text{CTDI}}_{\text{vol}}$, used to perform the CT examination.

Choices of X‐ray technique factors in CT imaging also determine the amount of radiation received by the patient.^4^, ^5^ The patient radiation dose is directly related to (any) patient radiation risk, which in head CT primarily relates to the possibility of a future malignancy. The most important goals in radiological imaging are, therefore, to ensure that patient benefits exceed any risks, and that unnecessary radiation is eliminated. To achieve these goals, the identification of the appropriate amount (and type) of radiation for a given type of radiological examination is of paramount importance. The optimum X‐ray beam quality and quantity will depend on both patient characteristics, as well as the specified diagnostic imaging task. For CT scans performed with iodinated contrast, it is customary to reduce the X‐ray tube voltage whenever possible, to maximize the visibility of the administered contrast material. For adult head CT scans the American College of Radiology CT Accreditation program requires that ${\text{CTDI}}_{\text{vol}}$, as measured in a small phantom (16 cm diameter), should not exceed 75 mGy, and that a ${\text{CTDI}}_{\text{vol}}$ of 80 mGy will result in a failed application.^(6)^

Current practice in radiological imaging frequently encounters wide variations of X‐ray technique factors both between institutions, as well as within a given institution. Reasons for these differences include variable preferences of radiologists and differences between how technologists perform examinations, as well as variations in vendor equipment design. A recent analysis of head CT protocols at our institution, which makes use of six CT scanners at three different physical locations, identified default protocol ${\text{CTDI}}_{\text{vol}}$ values with a maximum‐to‐minimum ratio of 25%. Although this situation is clearly suboptimal, there is little objective scientific data relating to how changes in radiation intensity affect perceived image quality or the corresponding diagnostic performance. This study made use of our existing images, obtained at a range of ${\text{CTDI}}_{\text{vol}}$ values, to identify how changing the amount of radiation used to perform routine head CT examinations affects the visibility of anatomical structure. The results helped identify the optimum CT technique factors for use when adult patients at our institution undergo head CT examinations.

## II. METHOD

### A. Head CT scanning at MUSC

This was a retrospective study aimed at investigating the relation between dose (${\text{CTDI}}_{\text{vol}}$) and diagnostic image quality for routine noncontrast head CT exams, conducted at our institution. Table 1 lists parameters and the six scanners used for performing diagnostic CT examinations. All images were acquired at 120 kV in a helical acquisition mode with a pitch between 0.55 and 0.9, a rotation time of 1 s with a fixed tube current between 300 mA and 500 mA, and a beam collimation of 0.6 mm±0.8 mm. Images were reconstructed with a head kernel (“standard” for GE, H40s for Siemens) at a slice thickness of 5 mm. No dose modulation (e.g., automatic exposure control) was used for any of the exams.

**Table 1:** CT scanners and the scan parameters used for performing routine head CT examinations.

*CT Vendor*	*CT Model*	*Rotation Time (s)*	*Pitch*	*Beam Collimation (mm)*	*mAs*	*Head* ${\text{CTDI}}_{\text{vol}}$ (mGy)
Siemens	Sensation 64	1.0	0.9	0.6	380	59.8
GE	LightSpeed	1.5	1.4	0.6	138	63.0
Siemens	Definition Flash	1.0	0.6	0.6	300	67.9
Siemens	Somatom Definition	1.0	0.7	0.6	500	71.8
Siemens	Sensation 16	1.0	0.9	0.6	350	73.6
Siemens	AS 128	1.0	0.7	0.6	400	74.7

${\text{CTDI}}_{\text{vol}}$ for a head CT examination is influenced by the specific choices made of mA, X‐ray tube rotation time, and helical pitch. Knowledge of mAs and pitch are insufficient to specify the X‐ray tube output in CT, which also depends on the X‐ray tube characteristics, X‐ray tube voltage, and beam filtration. By contrast, ${\text{CTDI}}_{\text{vol}}$ values in a specified phantom size and X‐ray beam quality provide a universal metric for quantifying the amount of radiation that is incident on any patient undergoing any type of examination. Table 1 also lists the ${\text{CTDI}}_{\text{vol}}$ (measured in a 16 cm phantom) that were used when performing routine head CT examinations at the time that this study was performed (i.e., January 2011 to April 2011).

### B. Patient selection

The study population included 28 females and 20 males (median age±σ=57 yr±17 yr;range=23 yr−84 yr). Images were selected from routine noncontrast head CT exams conducted in adult subjects on each of these six different scanners in the department. Eight exams were randomly chosen from each of these six scanners, resulting in a total of 48 normal patients. All exams chosen for the study were reported as being normal, without any evidence of underlying pathology.

**Figure 1:**
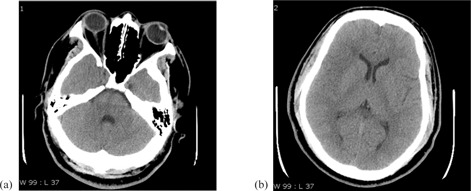
Representative head CT slices used in the study, at the level of (a) fourth ventricle, and (b) basal ganglia.

From each exam, two slices, one at the level of basal ganglia and one at the level of the fourth ventricle, were selected for evaluation. Figure 1 shows an example of images obtained at the fourth ventricle and basal ganglia. The number of images used in this study therefore consisted of 48 images at each of two anatomical regions (i.e., 6 scanners×8 exams). Images were gathered in a PACS database file for easy access to readers.

### C. Image Evaluation

Images were examined by three experienced neuroradiologists (11, 7, and 5 years' experience) from our department, on a standard reading room workstation. For the basal ganglia slice, the reader was asked to evaluate the differentiation of the putamen, caudate nucleus, thalamus, and the internal capsule. For the fourth ventricle slice, the reader was asked to evaluate the differentiation between grey and white matter, as well as the brainstem. Readers assigned an ineteger score ranging from 1 to 5 using the rating system depicted in Table 2.

Each reader was shown the complete dataset consisting of 96 images in a randomized manner and without the use of any patient demographic information or any other technique facts. Readers were allowed to change window and level settings as would be done during routine image interpretation. Scores were averaged over each slice location for each scanner for every reader. This yielded 12 average scores (6 scanners×2 anatomical locations), which were plotted as a function of the ${\text{CTDI}}_{\text{vol}}$ for each reader. In addition, scores from all readers were pooled and also plotted as a function of ${\text{CTDI}}_{\text{vol}}$.

**Table 2:** Reader scoring scheme that was used in this study.

*Reader Score*	*Interpretation*	*Comment*
1	Unacceptable	The image quality is so poor that an interpretation is not possible and the study would need to be repeated.
2	Barely satisfactory	The image is of poor quality; however, it answers the major clinical questions (Mass? Hemorrhage? Clear infarction?).
3	Satisfactory	The image quality is sufficient for adequate interpretation, however with clearly present artifacts and/or prominent noise, which may potentially obscure very subtle details.
4	Good	Better than average image quality with the noise and artifacts not even theoretically affecting diagnostic value.
5	Excellent	Outstanding image quality, free of artifacts and with imperceptible noise.

## III. RESULTS

**Figure 2:**
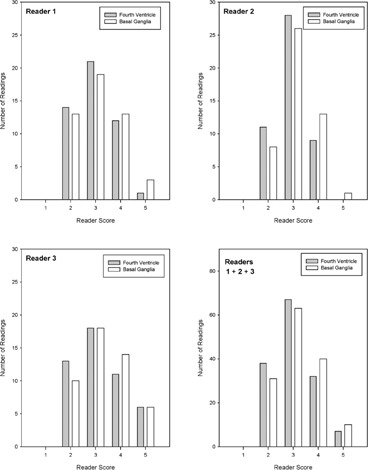
Histogram distribution of scores recorded for CT images of the fourth ventricle and basal ganglia.

Figure 2 shows the overall scores for all three readers viewing head CT images at the level of the fourth ventricle and basal ganglia. For CT images of the fourth ventricle, 26% received a score of 2, 47% a score of 3, 22% a score of 4, and 5% a score of 5. For CT images of the basal ganglia, 22% received a score of 2, 44% a score of 3, 28% a score of 4, and 7% a score of 5. Average scores for the fourth ventricle were 3.06±0.83 and for the basal ganglia were 3.20±0.86. No image received a score of 1 (unacceptable).

**Figure 3:**
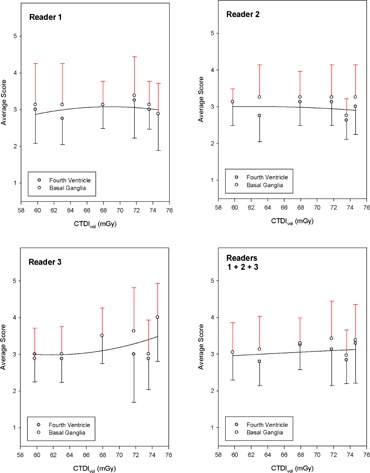
Average reader score as a function of the ${\text{CTDI}}_{\text{vol}}$ (mGy) that was used to generate CT images at the level of the fourth ventricle and basal ganglia.

Figure 3 shows the average reader scores plotted as a function of ${\text{CTDI}}_{\text{vol}}$ (mGy) used to generate each image. The closed circles relate to the fourth ventricle and the open circles relate to the basal ganglia. Solid lines show least squares fits to a second order polynomial. Error bars are the computed standard deviations for individual observers from scores of 48 images, with negative values pertaining to the fourth ventricle data and positive values to the basal ganglia data.

The data in Fig. 3 show that there are no consistent trends with increasing radiation dose used to generate these images. Readers 1 and 2 showed no clear trend of an increasing score with increasing ${\text{CTDI}}_{\text{vol}}$. Reader 3 showed a modest trend of an increased score with increasing ${\text{CTDI}}_{\text{vol}}$, but the magnitude of the observed increase in score when ${\text{CTDI}}_{\text{vol}}$ increased by 25% increase was markedly less than the measured standard deviations. Pooled reader data (Fig. 3) demonstrate that increasing the ${\text{CTDI}}_{\text{vol}}$ from 60 to 75 mGy had negligible effect on the overall average reader score.

## IV. DISCUSSION

The key to optimal imaging is to achieve the best possible balance between the diagnostic utility and patient safety. When ${\text{CTDI}}_{\text{vol}}$ is set too low, diagnostic performance could suffer because of increases in the false‐positive and/or false‐negative rates. A receiver operating curve (ROC) analysis would then be expected to show that reductions in the radiation intensity used to perform the head CT examinations (${\text{CTDI}}_{\text{vol}}$) result in a corresponding reduction in the area under the ROC curve ($A_{z}$). In addition, it is important to note that overzealous dose reductions could actually lead to an overall increase in radiation. A nondiagnostic exam could need to be repeated, which would obviously serve to increase the patient radiation dose. Diagnostic inadequacy should be the primary concern in any diagnostic examination, and is potentially more harmful than any overexposure.^3^, ^4^, ^7^ The reasons for this include increased false‐positives and false‐negatives, as well as the potential of unnecessary radiation exposure from repeat examinations that could easily result in a doubling of typical patient radiation exposures.

If the ${\text{CTDI}}_{\text{vol}}$ is set too high, there will be no drop in diagnostic performance, with the area under the ROC curve ($A_{z}$) unlikely to increase with increasing ${\text{CTDI}}_{\text{vol}}$. The problem with using ${\text{CTDI}}_{\text{vol}}$ values that are too high is that patients receive unnecessary radiation exposure.^7^, ^8^ The head is a relatively insensitive organ compared to the body, and typical head effective doses are of the order of 1 to 2 mSv, whereas those in body CT are typically in the range of 5 to 10 mSv.^9^, ^10^ Using a higher amount of radiation would likely be a better option than using too little radiation, because the potential harm to patients of the latter option are most likely to be much higher. However, very high ${\text{CTDI}}_{\text{vol}}$ in head CT examinations does not improve patient benefits, but only serves to increase the possible patient risks that pertain to the induction of cancer. Identification of the optimal amount of radiation is clearly the best option, and which was the motivation behind our study, because it is likely to maximize overall patient benefit.

Figure 2 indicates that the most frequent images score was 3 (satisfactory), with the remainder relatively evenly distributed between 2 (barely satisfactory) and 4/5 (good/excellent). Thus, there was no trend towards consistently high or low scores. In general, observers scored the images for basal ganglia higher than images for posterior fossa. This is understandable since differentiation of the basal ganglia, internal capsule, and thalamus is easier than the differentiation between grey and white matter at the level of the fourth ventricle, in addition to a relatively higher amount of streak artifact that is present in the posterior fossa.

Figure 3 shows how the CT radiation intensity (${\text{CTDI}}_{\text{vol}}$) affected the average reader scores. The nominal increase in observer score when ${\text{CTDI}}_{\text{vol}}$ increased from 60 to 75 mGy, based on the curve fits shown in Fig. 3 was about 0.2. By comparison, the average standard deviation for the 12 data points depicted in Fig. 3 was over four times higher than the apparent increase in image quality score by increasing ${\text{CTDI}}_{\text{vol}}$ by 25%. Of specific note is the fact that there was no consistent improvement in image quality score with increasing ${\text{CTDI}}_{\text{vol}}$ for all three observers, and that any increase in image quality score was always much lower than the standard deviation observed for a given observer at a constant ${\text{CTDI}}_{\text{vol}}$ value. The high standard deviation values that were obtained at any given ${\text{CTDI}}_{\text{vol}}$ value also suggest that radiation dose is a minor contributor to perceived image quality. For all these reasons, it is reasonable to conclude that there appear to be no benefits to be gained when ${\text{CTDI}}_{\text{vol}}$ increases from 60 to 75 mGy for routine head CT examinations performed in adult patients.

It is of interest to compare our “choice” of ${\text{CTDI}}_{\text{vol}}$ of 60 mGy with corresponding data in the scientific literature. The ACR CT accreditation values of ${\text{CTDI}}_{\text{vol}}$ for routine head CT examinations for the period 2002 to 2004 was 59.1±18.6 mGy. The 75th percentile value was 76.8 mGy, which resulted in an ACR reference value of 75 mGy for routine adult head CT examinations being introduced in January 2008.^(6)^ A survey of ${\text{CTDI}}_{\text{vol}}$ values for routine head CT examinations in the USA, UK, and Germany in the period 1999 to 2001 showed 59±6 mGy, with very little difference between European and US values.^(11)^ This same survey, however, showed that body ${\text{CTDI}}_{\text{vol}}$ values in the US were generally a factor of two higher than those that were being used in Europe. These data show an apparent consensus that routine head CT examinations should be performed using a ${\text{CTDI}}_{\text{vol}}$ of 60 mGy. Although we did not investigate doses that were below 60 mGy, the fact that most facilities around the world appear to be using this amount of radiation suggests that reducing doses is unlikely be warranted.

Patients with ventriculo‐peritoneal shunts for management of hydrocephalus often undergo multiple head CT examinations for assessment of shunt malfunction and it is this specific sub‐population of patients that are probably under the highest risk from head CT radiation effects. The assessment of shunt malfunction is most commonly done on an emergent basis when the alternative imaging modality (brain MRI) is not only more expensive but also frequently unavailable. Considering multiple head CT exams to which these patients are exposed, even a relatively modest decrease in dose of around 20% (${\text{CTDI}}_{\text{vol}}$ from 75 to 60 mGy in our study) is very beneficial, as it translates into one additional diagnostic head CT for the same cumulative dose of four exams with ${\text{CTDI}}_{\text{vol}}$ of 75 mGy (and many of these patients undergo more than 10 or even 20 head CT scans).

One limitation of the study is that the variability in ${\text{CTDI}}_{\text{vol}}$ values was not achieved by varying the parameters on the same scanner but rather by using different scanners. It is therefore possible that at least a portion of the observed differences (or the lack thereof) was due to variations in scanner design. This effect is likely small, but may be addressed in an investigation that would evaluate head CT scans with various ${\text{CTDI}}_{\text{vol}}$ (for example 60, 65, 70, and 75 mGy) on a single scanner. The other important component of the efforts to minimize unnecessary medical radiation exposure lies in elimination of exams that are not indicated, but overutilization of diagnostic imaging is beyond the scope of our study.

The most radiosensitive organs and tissues in the body are the lungs, red bone marrow, female breast, colon, and stomach.^(12)^ As a result, the head is generally considered to have a low radiation risk despite the fact that head CT scans use much more radiation than a corresponding body CT examination. Head CT scans have ${\text{CTDI}}_{\text{vol}}$ values that are typically double those of body CT scans when CT output is normalized to the same CT dosimetry phantom size, but have effective doses that are typically a factor of five lower. Nonetheless, it is still very important to minimize unnecessary radiation exposures for all patients. The risk of malignancy associated with ionizing radiation has been recently shown to be clearly more than a theoretical concern.^13^, ^14^, ^15^ Pearce et al.^(16)^ revealed that children receiving the radiation equivalent of five to ten head CT scans have an approximately three‐fold increase in relative risk of leukemia. The relative risk of brain tumor is tripled with only two to three head CT scans (~60 mGy brain dose). Understanding of patient doses and radiation risks in CT will thus continue to be important to ensure that CT scans result in a net patient benefit, and that unnecessary radiation is minimized by ensuring doses are As Low As Reasonably Achievable (ALARA).^17^, ^18^

## V. CONCLUSION

This study indicates that the visibility of important anatomical structures in routine head CT images is not improved when ${\text{CTDI}}_{\text{vol}}$ (dose) increases from 60 and 75 mGy. As a result of this study, all routine head CT exams at our institution are now conducted using a ${\text{CTDI}}_{\text{vol}}$ of 60 mGy.
